# Surprisal Analysis of Transcripts Expression Levels in the Presence of Noise: A Reliable Determination of the Onset of a Tumor Phenotype

**DOI:** 10.1371/journal.pone.0061554

**Published:** 2013-04-23

**Authors:** Ayelet Gross, Raphael D. Levine

**Affiliations:** 1 The Fritz Haber Research Center, Hebrew University, Jerusalem, Israel; 2 Department of Chemistry and Biochemistry, Crump Institute for Molecular Imaging and Department of Molecular and Medical Pharmacology, David Geffen School of Medicine California, Los Angeles, California, United States of America; University of Calgary, Canada

## Abstract

Towards a reliable identification of the onset in time of a cancer phenotype, changes in transcription levels in cell models were tested. Surprisal analysis, an information-theoretic approach grounded in thermodynamics, was used to characterize the expression level of mRNAs as time changed. Surprisal Analysis provides a very compact representation for the measured expression levels of many thousands of mRNAs in terms of very few - three, four - transcription patterns. The patterns, that are a collection of transcripts that respond together, can be assigned definite biological phenotypic role. We identify a transcription pattern that is a clear marker of eventual malignancy. The weight of each transcription pattern is determined by surprisal analysis. The weight of this pattern changes with time; it is never strictly zero but it is very low at early times and then rises rather suddenly. We suggest that the low weights at early time points are primarily due to experimental noise. We develop the necessary formalism to determine at what point in time the value of that pattern becomes reliable. Beyond the point in time when a pattern is deemed reliable the data shows that the pattern remain reliable. We suggest that this allows a determination of the presence of a cancer forewarning. We apply the same formalism to the weight of the transcription patterns that account for healthy cell pathways, such as apoptosis, that need to be switched off in cancer cells. We show that their weight eventually falls below the threshold. Lastly we discuss patient heterogeneity as an additional source of fluctuation and show how to incorporate it within the developed formalism.

## Introduction

Monitoring the changing expression levels of mRNAs and more recently miRNAs [1], [2] is carried out primarily for identification of disease and the response to treatment. One can probe the change in a cell culture as time changes or examine the variation among different patients, different environments etc with special reference to large data sets, e.g., [3]. We propose to use changing expression levels to obtain evidence for oncogenesis earlier in time before a cancer phenotype can be detected by more conventional means. The input that we require is transcription level of mRNAs measured at different points in time, spanning many cell divisions. The ongoing changes will be quantitated by surprisal analysis [4], a technique that integrates and applies principles of thermodynamics and maximal entropy towards the unbiased thermodynamic characterization of systems that change in time. Unlike clustering methods surprisal analysis determines first a base line, a state of maximal thermodynamic entropy. Once the system reaches its maximal entropy, it can no longer initiates or participates in spontaneous processes. The baseline pattern is very much the same in cells of different patients [5]. The Surprisal Analysis next determines sets of transcripts that collectively represent a deviation away from the reference state. Each such pattern is a signature of a process. All the transcripts in a given signature have a common variation with time. We determine these signatures from microarray or from deep sequencing data. It is found that very few, two, three four, processes suffice to quantitatively describe the expression levels of many thousands of transcripts. Our paper addresses the question of how do we know that we have extracted all but no more than all the information about the process that is contained in the data. Why is there an issue about no more than all? Because we are analyzing real experimental data and such data has always some noise. So it is not meaningful to provide a perfect fit of the data. Much of the effort in getting a perfect fit will be to fit the noise. In this paper we discuss the cutoff beyond which the identification of a phenotype is not reliable.

Clustering methods [6], [7] have been extensively and successfully used to seek significant patterns in microarray data. The method we use also groups transcripts into expression patterns with key differences. First, a pattern is not a cluster since a given transcript can belong to more than one pattern. Surprisal analysis is also not a statistical method because the grouping is based on assigning an inherent baseline weight to each transcript. This weight is thermodynamic-based. The measured expression pattern is profiled through the deviations from the base line. These deviations are small [5] because the base line reflects the cell machinery or ‘housekeeping’ genes [8]. The limited deviations from the base line means that detecting the weight of a disease pattern is numerically not straightforward. Lastly, our analysis determines the state of the cell and thereby enables us to predict the effect of a perturbation such as the addition of a drug [9].

Our paper provides both the basic theory and two illustrative applications to data from the laboratories of Varda Rotter [10], [11] and Alexander Levitzki [4], [12]. For both experiments we are able to demonstrate that rather suddenly and many cell divisions before a phenotype is evident, we detect the onset of a new process and the turning off of processes that can be identified with maintenance of healthy cells. It is the ability to put clear bounds on what is and what is not biologically warranted by the data that enables us to make categorical statements.

From cell lines we proceed to human patient cells in renal cancer using the data reported in Stickel [13]. The new feature in patient data is that quite typically the disease pattern is different in different patients [5]. The patient variability introduces an additional source of fluctuation in the data that must be taken into account.

The mathematical details are given in section S1 of Supporting Information S1. In the main text we give the sense of the derivations and the working results. Here we note that our theoretical considerations are based on the maximum entropy formalism [14]. For the time series data that we use this means that each time point the entropy of the transcription system is at the maximal possible value that is allowed by the constraints that act on the transcripts [4]. The constraints are imposed using the mathematical technique of the Lagrange multipliers [15]. The change of the expression level of the transcripts with time is represented through the Lagrange multipliers varying with time as shown in equation (1) below. The very large number of transcripts makes it convenient to use the Singular Value Decomposition (SVD) method [16] as a means of computing the Lagrange multipliers. SVD has been very effectively used in the analysis of microarray data [17], [18], [19], [20]. Here we use this mathematical technique in a different way and for us it is a method for effectively diagonalizing a non square matrix and thereby it provides [4] an efficient means of performing surprisal analysis. We also present an error analysis that takes advantage of features unique to the SVD procedure.

## Methods

We outline the theory that we developed and applied and we provide more details around the working results. In particular, the most practical form of the results is fully discussed. The notation used is that of surprisal analysis and this is introduced first. The role of patient variability is presented last. Mathematical details including those elements of Singular Value Decomposition, SVD, that are special to our application, are referred to the Supporting Information S1.

### Surprisal Analysis

The expression level of transcript *i* at time *t* is given by the procedure of maximal entropy as a fold change compared to the base line
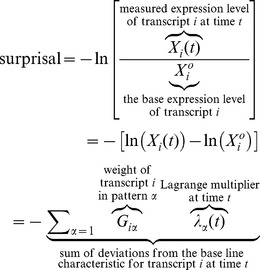
(1)


The fold difference is known as the surprisal. Surprisal analysis is the act of fitting of the surprisal by a sum of terms as shown in equation (1). There are typically very few terms that are needed in the sum. But exactly how many terms need to be included? This is the question addressed in this paper.

### The Data and the Error

Surprisal analysis consists in essence of the fitting of equation (1) to 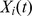 the measured expression level of transcript *i* at each time *t*. The best fit is sought by varying the values 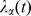 of the Lagrange multipliers. The practical way for minimizing the error is by using SVD as discussed in the SI and elsewhere (*4*). When expression levels are quantitated for example via a microarray the data is measured several times. The reading of the expression level of transcript *i* in different replicas are typically not quite the same. A *t* test is usually employed to reject such readings that differ too much between different replicas. But even those results that are kept after this test the different replicas do not quite yield the same level for a given transcript. This is the experimental error that we are discussing. The variability of different readings implies that the fitted values of the Lagrange multipliers will vary. It is the magnitude of this variation that we are after. The operational procedure that we will follow is to fit the Lagrange multipliers to the mean of the level of expression, mean over replicas. What we seek is the error bar on the value of each Lagrange multiplier.

### The Principle of Error Estimate in Surprisal Analysis

At each time *t* the importance of each term in the sum in the surprisal is determined by the value of the Lagrange multiplier 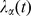 at that time. By inspection of equation (1) if the value of the Lagrange multiplier is zero, 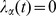, then that term is unimportant at that time and can be omitted.

One can state the conclusion about which constraint is important also in information theoretic terms: The value of the Lagrange multiplier is exactly by how much the constraint 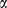 causes a lowering of the entropy from its global maximum, achieved at the base line. If at a time *t* we find that 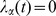 then the constraint 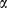 does not lower the entropy. In other words, at the time *t* constraint 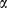 does not provide information on the state of the transcription system.

The first step is determining how many constraints are informative is to note that there can be no more than *T* where *T* is the number of measured time points. (This need not be a small number, see [21] for an example where *T* = 48). In general *T* is much smaller than the number *N* of transcripts. Even so it is shown in Supporting Information S1 that using the SVD method to diagonalize the covariance matrix with *T*−1 constraints and a baseline 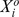 of *N* values one can reproduce the input data exactly.

It is the numerically perfect fit that the *T*−1 Lagrange multipliers provide that is the source of the issue we address in this paper. There is invariably some noise in the measured expression levels. So with *T*−1 Lagrange multipliers we fit both the real data and the noise. In this paper we estimate at what point the Lagrange multipliers begin to fit the noise [22].

The criteria we employ is direct: A Lagrange multiplier provides no additional information if its value is zero. So in the presence of noise, when there is an error range associated with each Lagrange multiplier, a Lagrange multiplier provides no new information when zero is a possible value. If 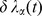 is the error range of the Lagrange multiplier for pattern 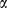 at the time *t*, then it is not informative at that time if
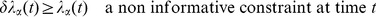
(2)


The remainder of the paper is how to determine the error bound on a Lagrange multiplier.

### The Constraints

In the maximum entropy formalism the numerical value of the Lagrange multipliers is determined by the mean value of the constraints. In terms of the time-independent variables 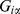 the mean value of the constraint 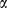 at the time t is given by
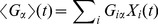
(3)


The time dependence of the mean value is due to the expression levels 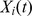 of the different transcripts that vary with time. The mean value 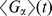 has an experimental error because the transcription levels 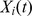 are only known to a finite accuracy that we denote as 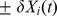. The prefix 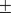 is because the sign of the error is not known and even more so, the correlation of the sign of the errors of different transcripts is not known. So we cannot compute the error in the mean value of 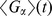 directly from its definition, i.e. using 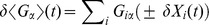. Note that in the expression for the error, 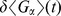, of the constraint we take it that the only source of error in the value of 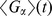 is due to the uncertainty in the expression levels meaning that there is no error in the values 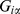 themselves. When we use SVD these values are determined from the data, (see Supporting Information S1), and so, potentially, there is another source of error.

### The Strict Upper Bound on the Error

Alhassid and Levine [23] have shown how to use the Schwarz inequality [24] as a practical way to compute an estimate of the error 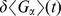 of the mean value of the constraint. There are a few differences between what we do here and the formalism used by Alhassid and Levine [23] These all stem from the fact that the sum of the expression levels does not have to equal unity nor need the sum be the same at different times. An adaptation of the method of [23] to the expression level data is discussed in the Supporting Information S1. The final result is an upper bound on the error of the Lagrange multipliers expressed in terms of the error measure *s* and a covariance matrix **M**

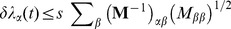
(4)


Here *s* is a (time-dependent) fold error that is summed over all expression levels
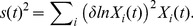
(5)



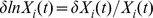
 is the fold error in the expression level of transcript *i* so that, for example, it equals 0.1 to represent an experimental error of 10%. If the fold error is about the same for all transcripts then 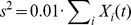 and note that in general 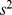 will scale with the total level of transcription, 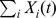. The elements 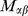 are the elements of the covariance matrix and are time dependent because the expression levels vary with time
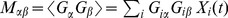
(6)


The upper bound given by equation (4) is a strict upper bound and it is the result we use when a careful analysis is required. But the computation requires inverting a matrix. So we turn next to a more accessible and practical expression that takes direct advantage of the use of SVD to diagonalize a matrix and thereby compute the surprisal expansion.

### The Practice of Error Estimate in Surprisal Analysis

When SVD is used to compute the surprisal [4] there is the advantage that the different deviation terms are orthogonal to one another, explicitly 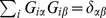 Here 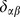 is the Kronecker delta symbol, 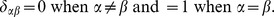 By using this in equation (1) we arrive at a practical expression for the Lagrange multiplier
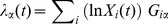
(7)where the time dependence is due to the expression levels. By virtue of this linear relation an error in the expression levels translates to an error in the Lagrange multipliers



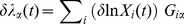
(8)Applying the Cauchy Schwarz inequality (*24*) to equation (8) we show in the Supporting Information S1 that
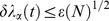
(9)where *N* is the number of measured transcripts and *ε* is the root mean square error
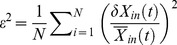
(10)


The practical error bound, equation (9), has the same value for all the constriants because it does not depend on the index 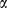. The output of the SVD procedure is usually arranged such that the constraints are listed in order of descending importance. Therefore, as a rough rule of thumb, one can expect that as the number of the constraints increases the constraints will become non informative. The reason that there can be exceptions is the following technical consideration. The SVD procedure gives the value of the Lagrange multiplier 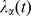 as
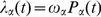
(11)where it is the eigenvalues 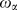 that are in descending order, 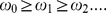 The time dependence of the Lagrange multiplier 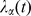 is given by the 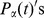 which can be viewed as components of a normalized eigenvector of the time-covariance matrix of the data, see [4] and Supporting Information S1.

### Early Time Forewarning of a Cancer Phenotype

At any time *t* equation (11) determines the value of the Lagrange multiplier at that time. To be informative at time *t* it is necessary that the error bound is low enough 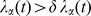. We are specifically concerned with such phenotypes 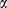 whose multiplier at very early times is very low and whose multiplier at late times is much higher. By very low and much higher we specifically mean that at very early times *t* and at later times we have that
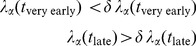
(12)The earliest time at which the phenotype 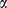 can be reliably said to contribute is when
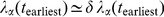
(13)which is the earliest time when the error is small enough.

There is a complementary situation for such phenotypes that are important in healthy cells and whose role gradually diminishes. For these we need to reverse the directions of the inequalities in equation (12). In the examples below the two boundaries coincide. Phenotypes that need to be switched off in cancer cells are no longer important at the same time range when the phenotype can be reliably discerned.

### The Role of Patient Variability

A source of noise that requires a separate discussion is when the data is not from a cell culture but represents an average over different patients. Using equation (1) for the Lagrange multiplier of a particular patient whose index is *m*, we define 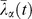 as the mean of 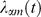 over the *M* different patients
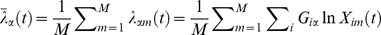
(14)From the mean and the individual 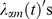 we can compute the statistical standard error of the Lagrange multiplier that is due to patient variability. Then 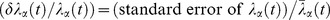
(15)


## Results

**Figure 1:**
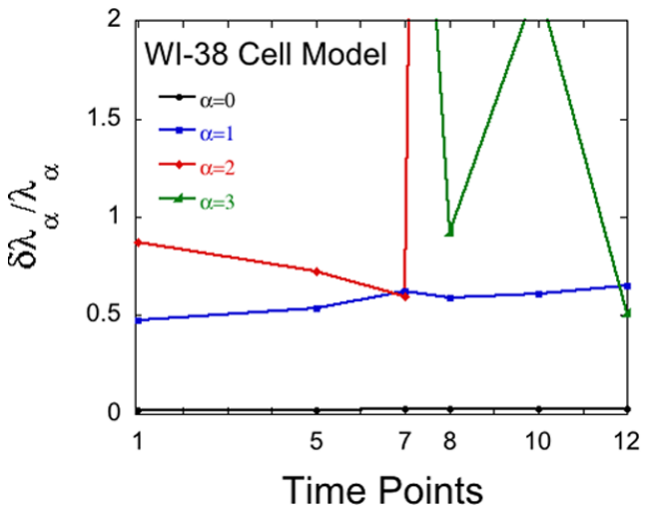
Reliability of weights of phenotypes during tumerogenesis. The upper bound on the fractional error in the Lagrange multipliers, 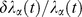, at different successive time points in the WI-38 cancer model of Rotter et al [11]. A constraint is warranted by the data when the fractional error is below unity, see equation (14). 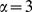 is the tumor signature and it is seen that it is only valid in later times but well before the cell is cancerous that is observed at time point 12. Note that the error in the steady state constraint is minimal.

The first example is the fractional error estimate in the Lagrange multipliers, 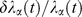, for steady state (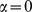) and the next 3 constraints 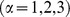 calculated using cell culture data for the WI-38 model developed by the Rotter group [11]. Surprisal analysis of the changes in the transcription pattern throughout the precancerous state identified three transcription patterns that suffice to reproduce the ternds in the expression levels. The major transcript, 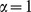, represents a contraction of signaling networks and an induction of cellular proliferation [10]. The fractional error for this constraint remains below unity at all measured time points, figure 1. The second constraint, 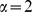, is seen in figure 1 to be meaningful only at the early time points. This pattern shows reduced expression of transcripts involved in cell cycle and cellular development [11]. Beyond the time point 7 the error for the 2^nd^ constraint (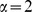) is already above the magnitude of the Lagrange multiplier itself 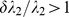.

The third constraint, 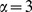, is the one that in references (*10,11*) was identified as the tumor signature pattern. It is seen in figure 1 that this constraint is only truly meaningful at the later times. We emphasize that also at ealier times the analysis yields a finite value for the Lagrange multiplier 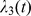, see figure 3 in [10] but the present error analysis shows that at these earlier times 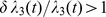 so that the constraint is not reliably warranted. A technical point of our work is this distinction between a finite but unreliable weight of the tumor signature pattern at early times and the sudden increase in reliability at intermediate times.

When using SVD it is a matter of notational convenience to represent the steady state as 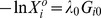. It is typically the case that 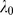 is far larger than the Lagrange multipliers so that, as seen in figure 1, the fractional error in 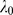 is quite small.

The bounds shown in figure 1 were computed using the strict upper bound given by equation (4). Using the more practical expression, equation (9) gives quite similar results.

**Figure 2:**
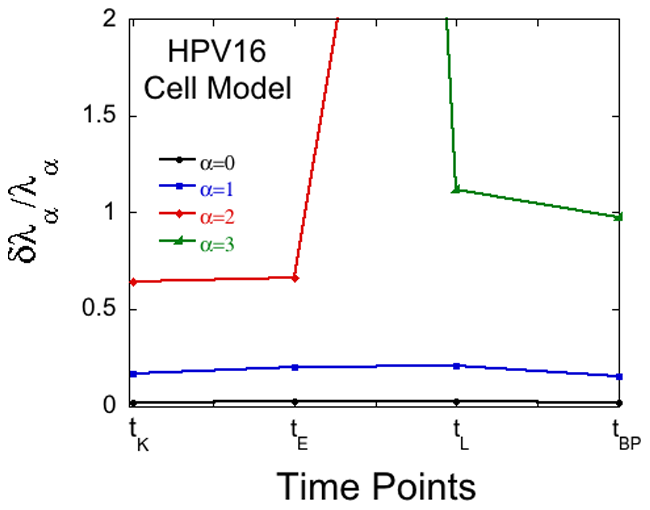
Soundness of weights of phenotypes during tumerogenesis. The bound for the fractional error of the Lagrange multipliers, 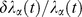, for steady state (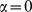) and the next 3 constraints 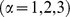 calculated for the four time points measured in the HPV-16 model [12].

The second example is the HPV-16 cancer model of Levitzki et al [12]. The results of the error analysis are shown in figure 2. There are four time points that were measured so that one can determine at most three constraints, (plus the base line makes four). It is seen in figure 2 that at any point in time there are only two constraints that are meaningful. The major one that is valid throughout, an early time one and a late time one. The late time constraint, 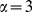, is identified as a tumor signature [4].

**Figure 3:**
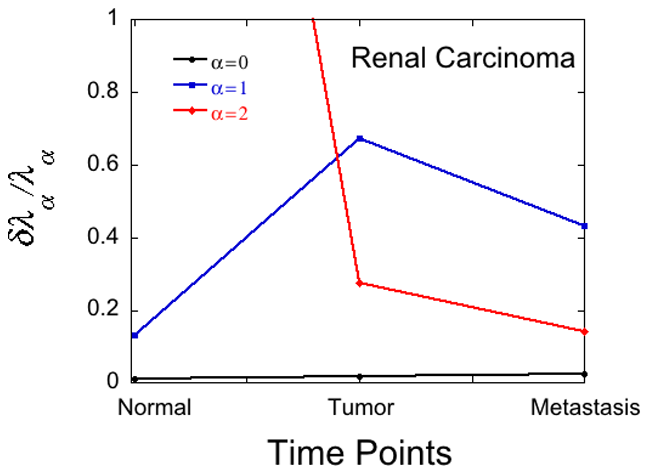
Reliability of weights of phenotypes for a renal cancer patient. The error bound in the Lagrange multipliers for steady state (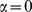) and the next 2 constraints 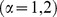 calculated for the 2^nd^ patient of renal carcinoma, using the data reported measured by Stevanović et al [13] for patient number 2. Quite similar results are obtained for the other two patients.

Lastly we consider the additional ‘noise’ due to patient variability. For each patient we can compute the Lagrange multipliers and their error due to noise in the measurements. Such results are shown in figure 3 for renal cancer at three time points as measured by Stevanović et al [13]. As in figures 1 and 2 also in the patient data in figure 3 we see a later in time phenotype becoming informative. It is informative for the diseased but not the healthy stage.

**Figure 4:**
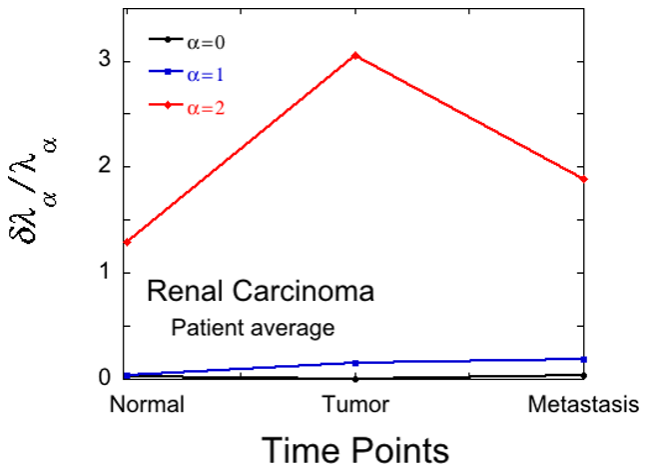
The importance of patient variability. The statistical error bound in the Lagrange multipliers for steady state (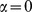) and the next 2 constraints 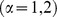 calculated taking into account patient variability by using equation (16). Renal cancer data for 3 patients as reported in Stevanović et al [13]. The error bound for the 2^nd^ constraint is high at all time points.

For each diseased patient separately we can use the renal cancer data of Stevanović et al [13] to determine reliably two constraints. But the disease signature of different patients are often quite different [5]. When one allows for this variability, using equation (15) of the results section, the late pattern is no longer reliable as shown in figure 4.

## Discussion

We analyzed transcription level changes over time in premalignant cell models and in cancer patients. A transcription pattern that is not expressed in healthy patients was seen in diseased patients. In early stage cells cultures an absent pattern was shown to become informative at later times. Later times but well before a cancer phenotype could be identified. Expressed or not expressed were judged on the basis of a conservative criterion based on an upper bound on the error in the weight of the transcription pattern. On both pragmatic and on information theoretic grounds it was argued that if the bound on the fractional error is below unity, the data warrants the conclusion that the phenotype is expressed. This suggest that with additional experience it could be possible to offer an earlier than currently possible diagnostics.

## Supporting Information

Supporting Information S1 (PDF)
