# Supplementary material for: Surprisal Analysis of Transcripts Expression Levels in the Presence of Noise: A Reliable Determination of the Onset of a Tumor Phenotype
Source: PLoS One. 2013 Apr 23;8(4):e61554. doi: 10.1371/journal.pone.0061554 (PMC3634025; doi:10.1371/journal.pone.0061554)
Supplement: Supporting Information S1 — (PDF) [file pone.0061554.s001.pdf]

Supplementary Information

Surprisal Analysis of Transcripts Expression Levels in the Presence of Noise: A Reliable Determination of the Onset of a Tumor Phenotype

A. Gross and R.D. Levine

In the Supplementary Information we provide details of the derivation of the error bounds and of the application of the SVD technique for the determination of the Lagrange multipliers. Throughout we refer to the expression levels as measured by averaging the readings over several replicas. By 'noise' or 'error' we mean that the readings of the different replicas are not identical. The fold error *s*, defined in equation (5) of the main text, is a measure of how much, on the average, do the values measured in individual replicas differ from one another.

S1. Derivation of the upper bound, equation (4) of the text

Using the maximum entropy formalism and the Lagrange multipliers technique we determine the distribution of expression level species *i* to have the functional form

$$\begin{aligned} \ln X_i(t) &= \ln X_i^o(t) - \sum_{\alpha=1} \lambda_\alpha(t) G_{i\alpha} \quad i = 1,2,...,N \\ &= -\sum_{\alpha=0} \lambda_\alpha(t) G_{i\alpha} \end{aligned} \tag{1}$$

where $X_i^o(t)$ is the expression level of species *i* in steady state, i.e., the distribution of expression levels at the thremodynamic maximum of the entropy. We show the steady state as being time dependent because in the computation we allow it to depend on time and we thereby have an internal consistency check because it must come out to be (nearly) time independent. For convenience we define $\ln X_i^o(t) = \lambda_0(t) G_{i0}$ and this allows us to write the second line in equation (1). Because we must be careful to count how many parameters note that there are *N* different values of $X_i^o(t)$ and there are *N*-1 independent values of G_{i0} because one value is determined by the normalization condition,

$\lambda_\alpha(t)$ is the undetermined Lagrange multiplier at time *t* for constraint α ($\alpha = 0$ being the steady state) and $G_{i\alpha}$ are the values of constraint α on transcript *i*, such that the observable $\langle G_\alpha \rangle$, the expectation value of constraint α, is defined as

$$\langle G_\alpha \rangle = \sum_i X_i G_{i\alpha} \tag{2}$$

In the application to transcription levels, whether of mRNAs or of miRNAs, it is possible to tell how many terms will have a Lagrange multiplier that has the value zero for all times. The reason is that the number of RNAs whose level can nowadays be measured is above a thousand for miRNAs and many thousands for mRNAs. Almost always we cannot measure for a similarly large number of times. So the index i of the RNAs is wider than the number of time points. Say that i varies from 1 to N and that the data is available at T different time points. So we have NT pieces of input data. Inspection of equation (1) shows that we need N different values of the $G_{i\alpha}$'s for each term in the sum. Unlike the situation in chemistry or physics we do not know a-priori the base line values so we have also N unknowns as the X_i^o's. We can therefore determine no more than T-1 Lagrange multipliers. This need not be a small number. A formal proof that also shows that there are no more than T-1 Lagrange multipliers that are not zero is provided in the supplementary information of (*2*). Furthermore, with T-1 Lagrange multipliers we obtain an exact recovery of the data. In other words, with T-1 Lagrange multipliers the right hand side of equation (1) is exactly equal to the right hand side for all transcripts i at all time points t.

In order to estimate the error we use equation (1) to write the variation of $\ln X_i$

$$\delta \ln X_i\,(t) = -\sum_{\alpha=0} \delta\lambda_\alpha(t) G_{i\alpha} \tag{3}$$

In principle $G_{i\alpha}$ is a property of species i and constraint α regardless of any noise in the data and therefore it does not change. In practice, in applications to large biomolecules we do not know the $G_{i\alpha}$'s from *ab initio* considerations. They need to be determined from the data. When one determines the eigenvectors using SVD as discussed in section S2, the $G_{i\alpha}$'s can be subjected to error except that as eigenvectors they are more resilient to error than the eigenvalues. This stability is a direct implication of the Rayleigh–Ritz method. Following equations (1) and (3), we can write the variation in $\langle G_\beta \rangle (t)$

$$
\begin{aligned}
\delta\left\langle G_\beta\right\rangle(t) &= \sum_i G_{i\beta}\delta X_i\,(t) \\
&= \sum_i G_{i\beta}X_i\,(t)\delta\ln X_i\,(t) \\
&= -\sum_{\alpha=0}\delta\lambda_\alpha(t)\sum_i\, G_{i\alpha}G_{i\beta}X_i\,(t) \\
&= -\sum_{\alpha=0}\delta\lambda_\alpha(t)\left\langle G_\alpha G_\beta\right\rangle(t)
\end{aligned} \tag{4}
$$

where we used the equality $\delta\ln x = \delta x / x$ and defined $\left\langle G_\alpha G_\beta\right\rangle \equiv \sum_i G_{i\alpha}G_{i\beta}X_i$.

Employing the Cauchy-Schwarz inequality ($\sum_k a_k b_k \le \left[\sum_k a_k^2\right]^{1/2}\left[\sum_k b_k^2\right]^{1/2}$), equation (4) can be rewritten as

$$
\begin{aligned}
\delta\left\langle G_\beta\right\rangle(t) &= \sum_i G_{i\beta}X_i\,(t)\delta\ln X_i\,(t) \\
&= \sum_i\left[G_{i\beta}\left(X_i\,(t)\right)^{1/2}\right]\left[\left(X_i\,(t)\right)^{1/2}\delta\ln X_i\,(t)\right] \\
&\le \left[\sum_i\left(G_{i\beta}\right)^2 X_i\,(t)\right]^{1/2}\left[\sum_i\left(\delta\ln X_i\,(t)\right)^2 X_i\,(t)\right]^{1/2} \\
&= \left\langle G_\beta G_\beta\right\rangle^{1/2} s
\end{aligned} \tag{5}
$$

where we define the fold error

$$
\begin{aligned}
s^2 &= \sum_i\left(\delta\ln X_i\,(t)\right)^2 X_i\,(t) \\
&= \frac{\sum_i\left(\delta\ln X_i\,(t)\right)^2 X_i\,(t)}{\sum_i\, X_i\,(t)}\sum_i\, X_i\,(t)
\end{aligned}.
$$

From equations (4) and (5)

$$
\begin{aligned}
&\sum_{\alpha=0}\delta\lambda_\alpha(t)\left\langle G_\alpha G_\beta\right\rangle(t) \le \left(\left\langle G_\beta G_\beta\right\rangle(t)\right)^{1/2}\cdot s \\
&\sum_{\alpha=0}\delta\lambda_\alpha(t)M_{\alpha\beta} \le \left(M_{\beta\beta}\right)^{1/2}\cdot s
\end{aligned} \tag{6}
$$

$M_{\alpha\beta}$ is defined as $M_{\alpha\beta} = \left\langle G_\alpha G_\beta\right\rangle$, which is the (α,β) element of the symmetric covariance matrix $\mathbf{M}$.

Consequently, the error estimation in the Lagrange multiplier of constraint α , λ_α , is

$$
\delta\lambda_\alpha(t) \le s\sum_\beta\,\left(\mathbf{M}^{-1}\right)_{\alpha\beta}\left(M_{\beta\beta}\right)^{1/2} \tag{7}
$$

Equation (7) offers an upper limit to the error in λ_α.

S2. Singular Value Decomposition. We determine the Lagrange multipliers by performing a Singular Value Decomposition, SVD, of a rectangular *N* by *T* matrix **Y** that is described below. The elements of this time dependent matrix are the surprisals of the transcription levels of the genes at different times, $Y_i(t) = \ln X_i(t)$. Each row corresponds to a particular transcript. Each column is at a particular value of time.

The SVD technique determines the non zero eigenvalues by diagonalizing a symmetric *T* by *T* matrix $\mathbf{Y}^{\mathrm{T}}\mathbf{Y}$ (The superscript T denotes the transpose of a matrix) This is an alternative to diagonalizing the much larger *N* by *N* matrix $\mathbf{Y}\mathbf{Y}^{\mathrm{T}}$ (*N* is the number of transcripts). The non vanishing eigenvalues of the two matrices are the same.

Since the entropy is a function of the (natural) logarithm of the expression level, $\ln X_i(t)$, we perform an SVD analysis over the logarithm of the measured expression level. The result of the SVD is an expression for the elements of the matrix **Y** (*1, 2*)

$$\begin{aligned} Y_{it} &= \ln X_i(t) \\ &= \ln X_i^o - \sum\nolimits_{\alpha=1} \lambda_\alpha(t) G_{i\alpha} \\ &= -\sum\nolimits_{\alpha=0} \lambda_\alpha(t) G_{i\alpha} \end{aligned} \tag{8}$$

Given the matrix elements Y_{it} we construct the symmetric $T \times T$ matrix $\mathbf{Y}^{\mathbf{T}}\mathbf{Y}$ that is diagonalized

$$\mathbf{Y}^{\mathrm{T}}\mathbf{Y}\mathbf{P}_\alpha = \omega_\alpha^2 \mathbf{P}_\alpha \quad \alpha = 0,1,2,\ldots,T-1 \tag{9}$$

The *T* numbers $\omega_\alpha(t)$'s are the eigenvalues of $\mathbf{Y}^{\mathbf{T}}\mathbf{Y}$, and the $\mathbf{P}_\alpha$ are the normalized eigenvectors. Because of the normalization, $\Sigma_i P_{i\alpha}^2 = 1$, the elements of **P** can only vary between -1 and 1. When we compare equation (8) with the result of the SVD of the matrix **Y** it follows that the Lagrange multipliers are given by (*1, 2*)

$$\lambda_\alpha(t) = \omega_\alpha P_\alpha(t), t = 1,2,\ldots,T \tag{10}$$

As *t* varies over the *T* data points, the values $P_\alpha(t)$ are the components of the eigenvector $\mathbf{P}_\alpha$. Because the size of the covariance matrix $\mathbf{Y}^{\mathrm{T}}\mathbf{Y}$ is *T* by *T* we can determine as many eigenvectors (or corresponding eigenvalues) as there are time points.

The Structure of equation (10) has important implications to the onset of a cancer phenotype or the turning off of a phenotype of a healthy cell, see equation (13) of the main text. Depending on how the components $P_\alpha(t)$ of a particular constraint α vary with time, the relevance of the constraint will vary. Figure 1 and 2 of the main text illustrate this point.

The eigenvalues ω_α's are arranged in descending order, thereby ω_0 being the first and largest eigenvalue, then ω_1 and so on. ω_0 is the eigenvalue of the steady state and is typically more than an order of magnitude larger than the other eigenvalues $\omega_{\alpha>0}$, (*1*). From equation (10) this implies that $\lambda_0(t) > \lambda_{\alpha>0}(t)$ at all times. Usually at a steady state $\lambda_0(t)$ does not change in time (*1-3*).

The singular matrix $\mathbf{Y}$ has a complementary set of eigenvectors $\mathbf{G}_\alpha$ that are determined by diagonalizing the *N* by *N* matrix

$$\mathbf{Y}\mathbf{Y}^T\mathbf{G}_\alpha = \omega_\alpha^2\mathbf{G}_\alpha \quad \alpha = 0,1,2,\ldots,N-1 \qquad (11)$$

The first *T* eigenvalues are identical to the *T* eigenvalues of the *T* by *T* matrix $\mathbf{Y}^T\mathbf{Y}$. See equation (9). All the other eigenvalues are identically vanishing. Numerically if one diagonalizes the matrix $\mathbf{Y}\mathbf{Y}^T$ the higher eigenvalues equal zero to within the numerical precision of the computer subroutine used for diagonalization. The elements of each one of the first *T* eigenvectors are the $G_{i\alpha}$'s $i = 1,2,..,N$ that are the values of the constraints as in equation (1) or (8).

To derive the practical bound, equation (9) of the main text, apply the Cauchy Schwarz inequality ($\sum_k a_k b_k \le \left[\sum_k a_k^2\right]^{1/2}\left[\sum_k b_k^2\right]^{1/2}$), on equation (8) of the main text resulting in

$$\begin{aligned}\delta\lambda_\alpha(t) &\le \left(\sum_i G_{i\alpha}^2\right)^{1/2}\left(\sum_i \left(\delta \ln X_i(t)\right)^2\right)^{1/2} \\ &= \left(\sum_i G_{i\alpha}^2\right)^{1/2}\left(\sum_i \left(\frac{\delta X_i(t)}{X_i(t)}\right)^2\right)^{1/2}\end{aligned} \qquad (12)$$

In this practical case the $G_{i\alpha}$'s are eigenvectors and thus normalized $\sum_i G_{i\alpha}^2 = 1$. Hence equation (12) can be rewritten as

$$\lambda_\alpha(t) \le \varepsilon\left(\sum_{i=1}^N 1\right)^{1/2} = \varepsilon(N)^{1/2} \qquad (13)$$

References

1. Kravchenko-Balasha, N., Levitzki, A., Goldstein, A., Rotter, V., Gross, A., Remacle, F., and Levine, R. D. (2012) On a fundamental structure of gene networks in living cells, *Proceedings of the National Academy of Sciences 109*, 4702-4707.
2. Remacle, F., Kravchenko-Balasha, N., Levitzki, A., and Levine, R. D. (2010) Information-theoretic analysis of phenotype changes in early stages of carcinogenesis, *Proc Natl Acad Sci USA 107*, 10324-10329.
3. Kravchenko-Balasha, N., Remacle, F., Gross, A., Rotter, V., Levitzki, A., and Levine, R. D. (2011) Convergence of logic of cellular regulation in different premalignant cells by an information theoretic approach, *BMC Syst Biol 5*, 42.
